# Diabetic Ketoacidosis and its Severity Predictors in Type 1 Diabetic Children; A 10-year Experience of A Teaching Hospital in Saudi Arabia

**DOI:** 10.1900/RDS.2022.18.146

**Published:** 2022-09-30

**Authors:** Waleed H. Albuali, Mohammad H. Al-Qahtan

**Affiliations:** Department of pediatrics, College of Medicine, King Fahad Hospital of the University, Imam Abdulrahman Bin Faisal University, Alkhobar, Saudi Arabia.

**Keywords:** diabetic ketoacidosis, type 1 diabetes mellitus, pediatric intensive care unit, precipitating factors, severity

## Abstract

**OBJECTIVE:**

Our objective was to determine the trend and precipitating factors of the severity of diabetic ketoacidosis (DKA) in the population admitted to the Pediatric Intensive Care Unit (PICU) in a large teaching hospital in the Eastern region of Saudi Arabia.

**METHODS:**

We conducted a retrospective, analytical study at King Fahad Hospital, Imam Abdulrahman Bin Faisal University, Alkhobar, Saudi Arabia. We retrieved the complete medical records of 2234 children who were admitted to the PICU during the 10-year period of 2010 through 2019. The children included those with polydipsia, polyurea, abdominal pain, vomiting, dehydration, and weight loss, as well as breathing disturbances due to acidosis and CNS issues such as lethargy or coma and elevated blood glucose level, > 200 mg/dL [> 11.1 mmol/L], venous pH 7.3, serum total CO2 15 mmol/L, and bloodhydroxybutyrate concentration 3 mmol/L or moderate or severe ketonuria.

**RESULTS:**

Out of 2234 PICU admissions, 211 (9.4%) were diagnosed with DKA. A persistent increase in the rate of DKA ended up at 14.1% in 2019 (p = .005). The incidence of DKA was 88/2234 (3.93%). The severity of DKA was as follows: 130 (61.6%) had severe and 81 (38.4%) had moderate DKA. Excessive sweet intake without adding insulin in 83 (39.3%) patients and unhealthy lifestyles (35.1%) were the best predictors of severe DKA (p = .001).

**CONCLUSION:**

Over a 10-year period, the DKA pattern was persistently rising and slightly falling, which ended up at the significantly highest rate of 14.1% in 2019. URTI, pneumonia, unhealthy lifestyle, and excess sweet intake were significant precipitating factors associated with severe DKA.

## 1 Introduction

Type 1 diabetes mellitus (T1DM) generally integrates diabetic ketoacidosis (DKA), that progresses to acute pediatric emergency. DKA characterizes a disorder due to acute metabolic disturbance following insulin deficiency to maintain metabolism of glucose in the body that contributes to high morbidity and mortality in T1DM pediatric age group [1]. The American Diabetes Association (ADA) and the World Health Organization (WHO) have established criteria for diagnoses of DKA [2]. An early diagnosis of DKA may be helpful for the patient as well as for the clinician to initiate appropriate treatment and prevent complications related to delayed diagnoses. Usually, new-onset T1DM is a common cause of DKA; however, preexisting T1DM, infection, another intervening disorder, or an underdose of insulin intake also may cause DKA [3,4].

The incidence of DKA in pediatric cohorts has been increasing worldwide. Its incidence in high-income countries like the United States is 4.8%, and about 6%-8% in central Europe, whereas it is 38.5% in Italy [5-7]. A systematic review of 31 countries reported rates of DKA between 12.8% and 80%; the United Arab Emirates and Saudi Arabia were seen with highest rates [8].

The major risk factors in newly diagnosed patients are younger age, family history of T1DM, poor socioeconomic class, lack of parental education, and less accessibility to medical services. For known T1DM patients, a lapse of insulin intake for one or more reasons, being peripheral to medical center resources because of their remote location, and improper insulin intake through a pump are the possible risk factors [9,10]. The common precipitating factors of DKA are abdominal pain, nausea, vomiting, fever, dehydration, polydipsia, polyuria, polyphagia, weight loss, drowsiness, loss of consciousness, tachypnea and deep and sighing respiration [11,12].

Pediatric diabetes mellitus is increasing in Saudi Arabia [13,14], and DKA is an associated complication of T1DM [15,16]. The massive economic growth and expanded digitalization over the past years in Saudi Arabia have brought adverse lifestyle behaviors, such as physical inactivity and intake of high-calorie sweetened products in children and youth, resulting in greater risk of diabetes mellitus and other noncommunicable diseases [17]. The objective of our study was to determine the latest trend of DKA and frequency of precipitating factors in relation to severity of DKA in a large teaching hospital in an eastern province of Saudi Arabia.

## 2 Methods

We retrieved complete medical records of 2234 pediatric patients who were admitted to the pediatric intensive care unit (PICU) during a 10-year period from 2010 through 2019. This study was conducted in King Fahd University Hospital (KFUH) of Imam Abdulrahman bin Faisal University (IAU), Alkhobar, Saudi Arabia. We collected signed consent forms from parents of pediatric patients prior to data collection.

The criteria for diagnosis of DKA were met if a child presented for the first time with combined symptoms of polydipsia, polyurea, abdominal pain, vomiting, dehydration, weight loss in addition to breathing disturbance due to acidosis and CNS issues like lethargy or coma and elevated blood glucose level > 200 mg/dL [> 11.1 mmol/L], venous pH < 7.3 or serum total CO_2_ < 15mmol/L, and blood β-hydroxybutyrate concentration ≥ 3 mmol/L or moderate or severe ketonuria and diagnosed with type 1 diabetes. The severity of DKA is categorized by the degree of acidosis [18]:

*Mild:* venous pH < 7.3 or serum bicarbonate < 15 mmol/L

*Moderate:* pH < 7.2, serum bicarbonate < 10 mmol/L

*Severe:* pH < 7.1, serum bicarbonate < 5 mmol/L

Children with missing or incomplete data in their files or with only hyperglycemia or who did not fulfill the criteria for DKA or with mild DKA were excluded from the study. The precipitating factors of DKA were assessed and diagnosed as follows:

Upper respiratory tract infection (URTI): screening of nasal aspirates and swabs.

Pneumonia: fever and breathing problem confirmed on chest radiograph.

Urinary tract infection (UTI): urine culture examination.

Missing dose and poor treatment compliance based on HbA1c > 6.4% mmol/mol.

Gastroenteritis following stomach and intestine inflammation, persistent lose motion, nausea and vomiting symptoms.

Unhealthy lifestyle following physical inactivity, drowsiness, and tired reflexes.

Excess sweet intake without insulin intake.

### 2.1. Data analysis

We used SPSS 22.0 (IBM, Armonk, NY) for data analysis. All categorical variables, including gender, severity and precipitating factors of DKA were presented as frequencies and percentages. We used the chi-square test for comparison of proportions of categorical variables in relation to the severity of DKA. To analyze the predictors of severe DKA in relation to the precipitating factors, we used logistic regression analysis by taking severity of DKA as a binary outcome variable and precipitating factors as covariates.

## 3 Results

**Figure 1.**
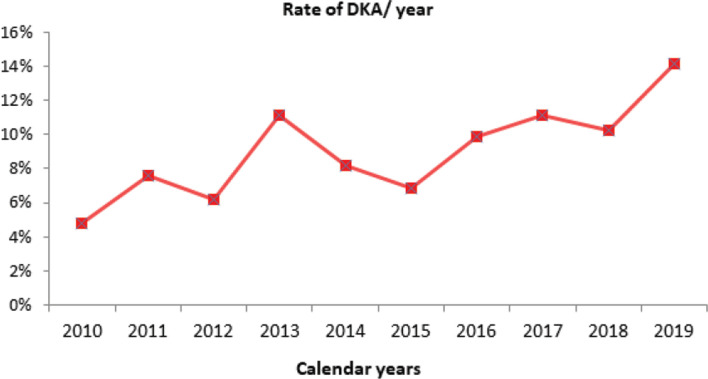
Rate of DKA per year. *Statistically significant at p = 0.005.

**Figure 2.**
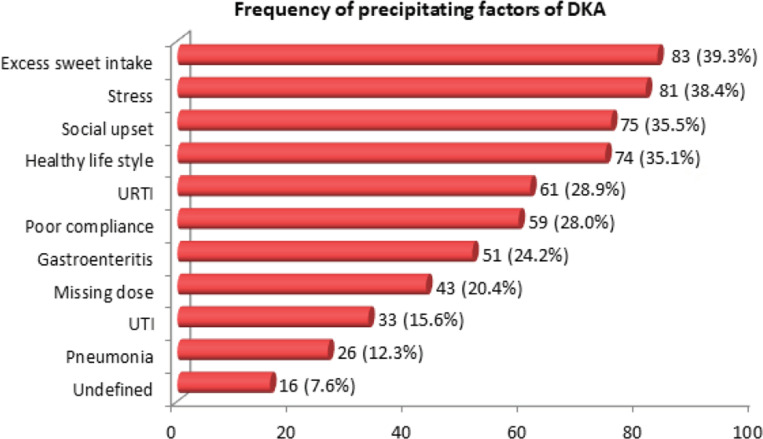
Frequency of precipitating factors of DKA

In the retrieved data during 10 calendar years from 2010 through 2019, a total of 2234 pediatric patients were admitted to the PICU. Of these, 211 (9.4%) were found to have DKA. From 2010 to 2013, the cases of DKA increased up to 11.1% (Table 1). After a slight decrease, it reached 11.1% again in 2017, and reached its significantly highest rate (14.1%) in 2019 (p = 0.005) as Figure 1 illustrates. Of the 211 patients with DKA, 123 (58.3%) were previously known cases, whereas 88 (41.7%) were newly diagnosed DKA. Thus, the incidence of DKA from PICU-admitted pediatric patients yielded 88/2234 cases (3.93%). Regarding the severity of DKA, out of 211 patients, 130 (61.6%) had severe and 81 (38.4%) had moderate DKA. Excess intake of sweets without additional insulin was the largest precipitating factor observed in 83 (39.3%) patients, followed by stress (38.4%), social upset (35.5%), unhealthy lifestyle (35.1%), URTI (28.9%), poor compliance to medication and follow-up (28%), gastroenteritis (24.2%), missing a medication dose (20.4%), UTI (15.6%), pneumonia (12.3%) and (7.6%) undefined (Figure 2). Precipitating factors associated with DKA severity were evaluated by using logistic regression analysis. The severity of DKA as moderate or severe was taken as binary variable, whereas gender and all precipitating factors were taken as covariates. URTI (OR = 4.0), pneumonia (OR = 41.3), social upset (OR = 7.1), healthy lifestyle (OR = 2.7), excess sweet intake (OR = 3.5) and stress (OR = 3.3) were the best predictors of severe DKA (p < 0.001), as Table 2 shows.

**Table 1.** 10-year trend of DKA

Years	PICU admissions (n = 2234)	DKA (n = 211)	Rate of DKA/year
2010	168	8	4.8
2011	158	12	7.6
2012	193	12	6.2
2013	135	15	11.1
2014	196	16	8.2
2015	248	17	6.9
2016	213	21	9.9
2017	234	26	11.1
2018	342	35	10.2
2019	347	49	14.1*
TOTAL	2234	211	9.4

* Statistically significant at p = 0.005

**Table 2.** Relationship of precipitating factors with severity of DKA

Factors	Categories	Severe (n = 130)	Moderate (n = 81)	OR (95% CI)	p-value
Gender	Male	80 (61.5)	40 (49.4)	1.6 (0.9-2.9)	0.083
	Female	50 (38.5)	41 (50.6)		
URTI	Yes	50 (38.5)*	11 (13.6)	4.0 (1.9-8.2)	< 0.001
	No	80 (61.5)	70 (86.4)		
Pneumonia	Yes	26 (20.0)*	0 (0)	41.3 (2.5-688)	< 0.001
	No	104 (80.0)	81 (100)		
UTI	Yes	16 (12.3)	17 (21.0)	0.53 (0.3-1.1)	0.091
	No	114 (87.7)	64 (79.0)		
Gastroenteritis	Yes	14 (10.8)	37 (45.7)*	0.14 (0.1-0.3)	< 0.001
	No	116 (89.2)	44 (54.3)		
Missing dose	Yes	24 (18.5)	19 (23.5)	0.74 (0.4-1.5)	0.381
	No	106 (81.5)	62 (76.5)		
Poor compliance	Yes	40 (30.8)	19 (23.5)	1.45 (0.8-2.7)	0.250
	No	90 (69.2)	62 (76.5)		
Social upset	Yes	65 (50.0)*	10 (12.3)	7.1 (3.4-15.0)	< 0.001
	No	65 (50.0)	71 (87.7)		
Unhealthy life style	Yes	56 (43.1)*	18 (22.2)	2.7 (1.5-5.1)	0.002
	No	74 (56.9)	63 (77.8)		
Excess sweet intake	Yes	65 (50.0)*	18 (22.2)	3.5 (1.9-6.5)	< 0.001
	No	65 (50.0)	63 (77.8)		
Stress	Yes	63 (48.5)*	18 (22.2)	3.3 (1.8-6.2)	< 0.001
	No	67 (51.5)	63 (77.8)		
Undefined	Yes	7 (5.4)	9 (11.1)	0.46 (0.2-1.3)	0.126
	No	123 (94.6)	72 (88.9)		

## 4 Discussion

Our results exhibited rising and slightly falling rates of DKA that ended at the significantly highest rate of 14.1% in 2019 (p = 0.005). The overall rate of DKA was 9.4%. Comparing the rate of DKA in pediatric diabetes mellitus in our study was considerably less than the reported rates of DKA in European countries including Germany, Italy, Romania and the United Kingdom 24%-67%. In our neighboring country of Kuwait, it was 49% [19,20].

In our study, the incidence of DKA from PICU-admitted pediatric patients yielded a rate of 88/2234 (3.93%) that is consistent with the incidence range 0.8%-5.6% reported in a systematic review based on 19 studies, in which 8 studies reported incidence of DKA in high income counties including North America, Europe and Israel [21]. In the same systematic review, the prevalence of DKA was reported by 11 studies with ranges from 0% to 12.8%; the author reported that prevalence was decreasing with increased age. In our study, we could not calculate prevalence of DKA due to our retrospective data collection and absence of having a denominator to identify number of pediatric patients from the registry in the specified period to be screened for diagnosis of DKA. In a local study done in Al-Baha region, the reported prevalence of T1DM was 355 per 100,000 population, but prevalence of DKA was not reported, although the frequency of DKA was 192, in which 19.8% were severe cases, 53.1% were moderate, and 27.1% were mild – findings contrary to ours where the majority of DKA cases (61.6%) were severe, and 38.4% were moderate. In our study, excess intake of sweets without adding insulin was the highest precipitating factor (39.3%) followed by stress (38.4%), social upset (35.5%), healthy lifestyle (35.1%), URTI (28.9%), poor compliance (28%), gastroenteritis (24.2%), missing dose (20.4%), UTI (15.6%), pneumonia (12.3%) and (7.6%) undefined. Other studies have reported similar precipitating factors and proportions [13,23]. However, Sonwari et al [23] additionally reported precipitating factors associated with mortality in DKA, as the majority of them had diabetes for a duration > 10 years (23.1%) (p = 0.012); for those with poor compliance (10.2%) (p = 0.028), those with cardiovascular disease (40%) (p = 0.004), or acute coronary syndrome, i.e., ACS (40%) (p = 0.004), DKA created a life-threatening state. A study reported 37.7% incidence of DKA among 147 newly diagnosed T1DM pediatric patients, and significant cases of moderate to severe DKA [24]. Likewise, the incidence of DKA and the prevalence of DKA in T1DM diagnosis ranged widely across national borders worldwide, and multiple factors were implicated, depending on the nature of population and healthcare facilities available [25]. A local study [26], reported 2 significant predictors: (1) healthy lifestyle without dietary and glycemic control was 29 times more likely and (2) excess intake of sweets without insulin showed a 3.31 adjusted hazards ratio. Our study is consistent with the reported study [26], poor glycemic control with healthy lifestyle (OR = 2.7) and excess sweet intake (OR = 3.5); in addition, URTI (OR = 4.0), pneumonia (OR = 41.3), social upset (OR = 7.1) and stress (OR = 3.3) were significant predictors of severe DKA (p < 0.001). Usher-Smith et al reported inverse association of frequency of DKA with gross domestic product, unlimited autonomy in lifestyle and dietary intake, and background incidence of T1DM [27]. It indicates a need for complete care and strict vigilance of T1DM patients for compliance of treatment, dietary recommendations, regular physical activity, and immediate access to a healthcare facility in case of any irregular event. In established T1DM patients, DKA recurrence may be more likely to reveal a positive family history of DM with an emphasis on general awareness and community education programs for formerly unidentified populations at high risk of DKA [10]. Pediatric diabetes awareness programs and screening campaigns at schools should be administered on a periodic basis to target and educate youth and their parents for special care in childhood that may minimize the incidence of DKA and promote early diagnoses. Furthermore, general awareness also should be enhanced for prevention, early detection of DKA among youth, and promotion of a healthy lifestyle consistent with high productivity and national development needs.

### 4.1 Study limitations

One of the limitations of our study was our inability to identify the exact magnitude of patients’ insulin daily doses per kg of body weight, thereby confirming the appropriateness of the insulin dose. A further limitation of our study was the unavailability of data related to social factors, including parental education – specifically, their care and role in compliance with glycemic control in T1DM that may have the potential to cause confounding effects in development of DKA in the pediatric population. We recommend further cohort studies to evaluate the effect of known versus new DKA cases, including biochemical profiles and clinical characteristics.

### 4.2 Conclusion

We found rising and then falling rates of DKA that ended up closing out the 10-year period at a rate significantly higher than any other time during the decade under study (14.1% in 2019). The intake of sweets without adding insulin, and an unhealthy lifestyle were the best predictors of severe DKA, consistent with the factors reported in existing literature. In addition to these 2 factors, our work also focused attention on URTI and pneumonia as significant precipitating factors associated with severe DKA.

## Abbreviations

T1DM: Type 1 diabetes mellitus

DKA: Diabetic ketoacidosis

WHO: World Health Organization

PICU: Pediatric intensive care unit

CNS: Central nerves system

URTI: Upper respiratory tract infection

ACS: Acute coronary syndrome
